# Universality class of explosive percolation in Barabási-Albert networks

**DOI:** 10.1038/s41598-019-44446-2

**Published:** 2019-06-13

**Authors:** MD. Habib E Islam, M. K. Hassan

**Affiliations:** 1 University of Dhaka, Department of Physics, Theoretical Physics Group, Dhaka, 1000 Bangladesh; 2 Old Dominion University, Department of Physics, 4600 Elkhorn Ave Norfolk, VA 23529 USA

**Keywords:** Condensed-matter physics, Statistical physics, thermodynamics and nonlinear dynamics, Phase transitions and critical phenomena

## Abstract

In this work, we study explosive percolation (EP) in Barabási-Albert (BA) network, in which nodes are born with degree *k* = *m*, for both product rule (PR) and sum rule (SR) of the Achlioptas process. For *m* = 1 we find that the critical point *t*_*c*_ = 1 which is the maximum possible value of the relative link density *t*; Hence we cannot have access to the other phase like percolation in one dimension. However, for *m* > 1 we find that *t*_*c*_ decreases with increasing *m* and the critical exponents *ν*, *α*, *β* and *γ* for *m* > 1 are found to be independent not only of the value of *m* but also of PR and SR. It implies that they all belong to the same universality class like EP in the Erdös-Rényi network. Besides, the critical exponents obey the Rushbrooke inequality *α* + 2*β* + *γ* ≥ 2 but always close to equality.

PACS numbers: 61.43.Hv, 64.60.Ht, 68.03.Fg, 82.70.Dd.

## Introduction

Percolation is still studied *in extenso* even after more than 60 years of its first formulation^1–7^. One of the reasons is that its notion is omnipresent in systems as disparate as spread of forest fire, flow of fluid through porous media, spread of biological and computer viruses leading to epidemic, formation of public opinion, resilience of systems etc.^8–11^. However, the most important reason is that it can describe phase transition and critical phenomena albeit it requires no quantum and many particle interaction effects into consideration^12^. To study percolation we first need to choose a skeleton. Traditionally, physicists like spatially embedded lattices as a skeleton. On the other hand, prior to 1999 random graph, namely Erdös-Rényi (ER) network, has also been used as a skeleton to study percolation though mostly by mathematicians^13^. In recent years use of network as a skeleton has gained huge interest among scientists in general and physicists in particular owing to two seminal papers of which one on small-world network and the other on scale-free (SF) network^14–16^. The idea of scale-free networks proposed by Barabási and Albert has revolutionized the notion of the graph theory. One of the hallmark features of the SF network is the presence of hubs which is hold responsible for making the SF network highly resilient to random removal of links albeit remains vulnerable to targeted removal^17,18^. The resilience to random attack is in a sense opposite to the process of percolation. In percolation one studies the conditions leading to the formation of the giant, a cluster of nodes connected by occupied links that grows linearly with network size *N*, while in the resilience one checks the condition of the disappearance of the giant.

In 2009, Achlioptas *et al*. proposed a simple variant of the classical random percolation (RP) and applied it in the ER network^19^. The skeleton of the ER network consists of *N* isolated nodes and *N*(*N* − 1)/2 number of frozen links among all the pairs of *N* nodes. Then at each step two distinct frozen links are picked at random instead of one which is done in RP. However, ultimately only one of two links, that suppresses the growth of the larger clusters, is activated and occupied while the other link is discarded for recycle. A specific rule that discourages the growth of the larger cluster and encourages the smaller ones to grow faster is the now well-known Achlioptas process (AP). It has been found that such a simple change in the selection rule proved to cause a dramatic effect in the final outcome. Indeed, as we keep occupying links following the rules of the AP, we find the emergence of a giant cluster with a bang. This is in sharp contrast to its classical counterpart and hence it is called “explosive percolation” (EP). Indeed, the corresponding order parameter *P*, the relative size of the largest cluster *s*_max_/*N*, undergo such an abrupt transition that it was at first mistaken as a discontinuity and suggested to exhibit the first order or discontinuous transition. This claim immediately triggered an explosion of scientific activities^20–29^. To this end, it is now well settled that the EP transition is actually continuous but with some first order like finite-size effects^28–31^.

Recently, it has been reported that RP in scale-free weighted planar stochastic lattice (WPSL) belongs to a universality class which is different from the unique universality class of all the known planar lattices^32,33^. Earlier, Cohen *et al*. reported that the universality class of RP on scale-free networks is different from the expected universality class of RP on infinite dimensional ER or Bethe lattice^34^. Motivated by the role that the scale-free nature of the skeleton plays in percolation, we study two variants of EP, namely product rule (PR) and sum rule (SR) under AP, on scale-free BA networks whose nodes are born with degree *m*. We then address the following questions: Does the PR-SR universality that holds in the ER network also hold in the BA network? Does EP in the ER and BA networks belong to the same universality class as expected since ER and BA networks both are infinite dimensional? If not, does EP in the BA network still obey Rushbrooke inequality? Besides addressing these fundamental questions, we find the following results. The critical points depend on *m* but not the critical exponents. We attempt to explain the significance of order and disorder in percolation using the idea of entropy and order parameter. We find that, like ferromagnetic transition, percolation too is accompanied by order-disorder transition.

The rest of the article is organized as follows. In section Sec. II we discuss some of the properties of the BA network to see how they differ with *m*. In Sec. III, the explosive percolation model is defined and the idea of convolution is introduced. Sec. IV contains definitions and properties of entropy and order parameter. In Sec. V we present our results on specific heat and susceptibility. In Sec. VI on the other we discuss about the scaling properties and the Rusbrooke inequality. The results are discussed and conclusions drawn in Sec. VII.

## BA Model and its Properties

**Figure 1:**
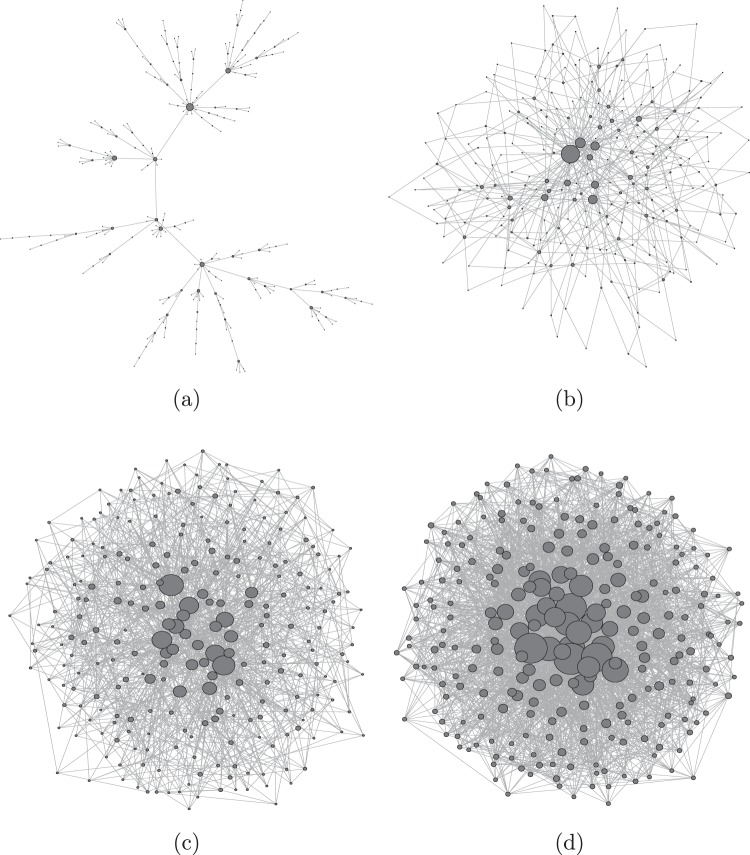
Snapshots of BA networks for *m* = 1, 2, 5, 10 consisting of *N* = 256 nodes are shown to appreciate the impact of *m* even in the impression. The size of the nodes are depicted as being proportional to their respective degree.

We begin by discussing the construction process of the BA network. It starts by choosing a seed that may consists of *m*_0_ arbitrarily connected nodes where *m*_0_ has to be extremely small compared to the final size *N* of the network that we intend to grow. However, it has been found that the observable quantities are independent of the size of *m*_0_ and of the detailed nature of how nodes of the seed are connected. Once a seed is chosen, we add one node with *m* links at each time step, where *m* ≤ *m*_0_, and pick *m* distinct nodes from the existing network such that node *i* is picked with probability Π(*i*) proportional to its own degree *k*_*i*_. It has been shown by BA that the network that grow following this preferential attchment (PA) rule, Π(*i*) ∝ *k*_*i*_, exhibits power-law degree distribution $P(k) \sim {k}^{-\lambda }$ with exponent *λ* = 3 indeoendent of *m*. The total number of links in the BA networks of size *N* is approximately equal to *mN* since each node is born with degree *m*. Each link contributes to degree 2. Thus the total degree is equal to 2 *mN* and hence the average degree of the BA network of size *N* is 2 *m* and hence for *m* = 1 it is a tree network. Network grown in this way can be used as a skeleton provided the snapshots of the network in the same realization at different times are similar. In ref.^35^ we have shown that the snapshots of the BA network at different times are similar and hence we say it exhibits self-similarity. In Fig. (1) we give snapshots of the BA network consisting of 256 nodes grown for *m* = 1, 2, 5, 10 just to grasp how the value of *m* keep its signature even in their visual look.

**Figure 2:**
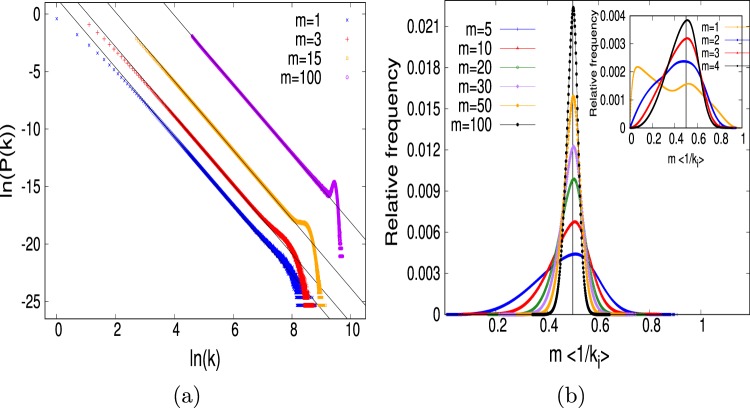
(**a**) Degree distribution of the BA network for different *m* value. (**b**) Plots of the relative frequency distribution of the IHM value as a function of IHM.

The question is: Do all the data points follow the power-law decay of *P*(*k*) with the same exponent? In Fig. (2a) we show how the degree distribution varies with *m*. We can clearly see that the number of data points for small *m*, especially the data points for small *k*, do not fall on the straight line of the log(*P*(*k*)) versus log(*k*) plot. However, as the *m* value increases the trend to follow the straight line increases. Indeed, we find that already at *m* = 100 it appears that almost all the data points, except the finite-size effect, follow the straight line which has slope equal to 3. Moreover, we observe that the extent of straight line increases with increasing network size revealing that in the thermodynamic limit it will be a straight line all the way through. The impact of *m* in the growing network can be best understood by measuring the inverse harmonic mean (IHM) of the neighbours of each node^36^. The value of IHM of the *i* th node is defined as

1 $${{\rm{IHM}}}_{i}=\frac{\sum _{j=1}^{{k}_{i}}\,\frac{1}{{k}_{j}}}{{k}_{i}},$$

where *k*_*i*_ is the number of nearest neighbors of the node *i*. If we now measure the fraction of the nodes which have IHM values within a given class, which we call relative frequency, then the resulting histogram plot for different *m* value is shown in Fig. (2b). It clearly shows the strong impact of *m* in the plots. The plots for higher *m*, such as *m* = 70 or 100, seem very different from those for the smaller *m* such as for *m* = 1 or *m* = 2 etc. Thus, the BA network for different *m* is significantly different albeit the exponent of the degree distribution is the same.

## Definition of Explosive Percolation

Once the network of desired size is grown we use that as a skeleton like we earlier used scale-free weighted planar stochastic lattice and ER network^37–39^. We assume that all the links of the BA network are temporarily frozen. We first label all the frozen links so that *e*(*i*, *j*) represents a link that establishes a connection between nodes *i* and *j* ensuring that *i* ≠ *j* since self connection is forbidden. Earlier, two groups independently studied EP to form scale-free networks instead of using a scale-free network as a skeleton^22,25^. In contrast, we use the scale-free BA network as a skeleton from where we pick a pair of candidate links randomly at each step and follow the rest of the process exactly like Achlioptas *et al*.^19^. Links can be of two types. When occupation of a link connects two isolated clusters then it results in a larger cluster and it is called inter cluster link. On the other hand, when a link connects two sites of the same cluster, which does not result in the increase in the cluster size, then the corresponding link is called intra-cluster link. Initially, every node is a cluster of its own size since all the links are assumed frozen. The process starts by picking two distinct links, say *e*(*i*, *j*) and *e*(*k*, *l*), randomly with uniform probability at each step. To apply the PR, we then calculate the products, Π_*ij*_ = *s*_*i*_ × *s*_*j*_ and Π_*kl*_ = *s*_*k*_ × *s*_*l*_, of the size of the clusters that the two nodes on either end of the links *e*(*i*, *j*) and *e*(*k*, *l*) respectively contain. The link with the smaller value of the products Π_*ij*_ and Π_*kl*_ is occupied; in case of Π_*ij*_ = Π_*kl*_ one of the corresponding links is selected randomly. On the other hand, to apply the SR, we take the sum Σ_*ij*_ = *s*_*i*_ + *s*_*j*_ and Σ_*kl*_ = *s*_*k*_ + *s*_*l*_ instead of the product and do the same as for PR.

One of the problems with percolation on network is the absence of the spanning cluster that spans from one end to the other end across the system since there is no boundary or end in network. Therefore a definition of spanning probability for percolation on network is not possible; this in turn makes it a formidable task to find the critical point and the critical exponent *ν* of the correlation length accurately. Yet another problem in percolation in general is that we need to perform many independent realizations. It works fine if the observable quantity, say *X*, is obtained directly from the ensemble averaged data. However, sometimes we may need to take derivative of *X* to obtain say *Y* = *X*′. In such cases, the ensemble average of *X* does not give smooth enough data for *Y* regardless of how big the ensemble size is. The only solution then is to use the idea of convolution as prescribed by Newman and Ziff in 2000^40,41^. According to their approach, we first need to measure the observable, say *X*_*n*_, as a function of the number of occupied links *n*. Note that we continue the process of occupying links *n* until *n* = *N* links are occupied or relative density of link *t* = *n*/*N* = 1 is achieved. We now take an ensemble averaged data over many independent realizations. We then use the resulting data in the convolution relation

2 $$X(t)=\sum _{n=1}^{N}\,{t}^{n}{(1-t)}^{N-n}{X}_{n},$$

to obtain *X*(*t*) for any value of *t*. The appropriate weight factor for each *n* at a given *t* is ${\sum }_{n=1}^{N}\,{t}^{n}{(1-t)}^{N-n}$^40,41^. The convolution relation takes care of that weight factor and hence helps obtaining a smooth curve for *X*(*t*) as a function of *t*. Its importance can be best appreciated when one takes the derivative of *X*(*t*) and then plot *dX*(*t*)/*dt* as a function of *t*. The resulting plot will be far too smooth compared to the ones without convolution which cannot be compensated by increasing the ensemble size to obtain ensemble averaged data.

## Entropy and Order Parameter

Entropy *H* and order parameter *P* definitely are the two most important quantities of interest in the theory of phase transition as they are used to define the order of transition. For instance, if they suffer a sudden jump or discontinuity at the critical point then the transition is first order and else it is called continuous or second order phase transition. Besides, they are also used as a litmus test of whether the transition is accompanied by symmetry breaking or not. In the case of symmetry breaking the system undergoes a transition from the disordered state, characterized by maximally high entropy, to the ordered state, characterized by maximally high order parameter. Note that the highly ordered state is always less symmetric than the highly disordered state. For instance, in ferromagnetic to paramagnetic transition we find that *P* and *H* undergo such an abrupt or sudden change but without gap or discontinuity at *t*_*c*_. In the case of percolation, it has been well known that the order parameter changes in the same fashion as in ferromagnetic transition. However, to know whether percolation transition too is accompanied by symmetry breaking or not we have to know how entropy changes at the critical point. Only recently we proposed a suitable normalized probability and obtained desired behaviour of entropy for the first time by using it in the definition of Shannon entropy

3 $$H(t)=-\,K\sum _{i}^{m}\,{\mu }_{i}\,\mathrm{log}\,{\mu }_{i},$$

where we choose *K* = 1 since it merely amounts to a choice of a unit of measure of entropy^42^. Surely one can use any normalized probability in Eq. (3) and claim to have measured entropy. Especially, being percolation a probabilistic model, there is no short of normalized probability. Note that originally in the information theory, the sum in Eq. (3) is taken over individual labelled message. In percolation, we regard each cluster as equivalent to message hence the sum cannot be over the size of the clusters rather over the individual cluster label. In fact, none of the existing probabilities which we know in percolation theory can be used to measure entropy although there have been some attempts^43,44^.

**Figure 3:**
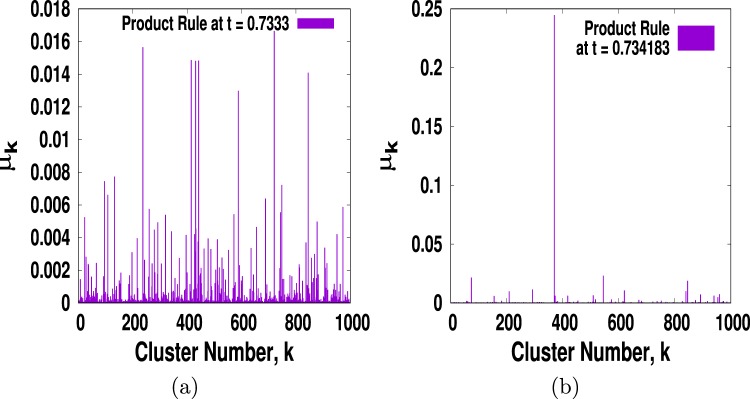
Plots of cluster picking probability *μ* as a function of cluster label *i* to show abruptness of its change as *t* changes from (**a**) *t* = 0.7333 to (**b**) *t* = 0.7341. It clearly shows that near *t*_*c*_ the giant cluster emerges within an extremely small change in *t*.

We assume that there are *m* distinct, disjoint, and indivisible labelled clusters *i* = 1, 2, ..., *k* whose sizes are say *s*_1_, *s*_2_, ...., *s*_*k*_ respectively after addition of *n* = *tN* links. To measure entropy for percolation we use the Shannon entropy where we choose *μ*_*i*_ = *s*_*i*_/*N* as the cluster picking probability that a site being picked at random will belong to the cluster *i*^32,33,37^. Note that initially each node is a cluster of its own size *s* = 1 and hence *μ*_*i*_ = 1/*N* for all the nodes *i* = 1, 2, ..., *N*. This is exactly like the state of the isolated ideal gas where all allowed microstates are equally likely and hence it is expected that entropy is maximum at *t* = 0. To see what happens at the other extreme, at *t* = 1, we first plot histogram of *μ*_*i*_ versus cluster label *i* in Fig. (3) for PR with *m* = 5 only to show what happens to the probabilities {*μ*_1_, *μ*_2_, ..., *μ*_*k*_} in the vicinity of *t*_*c*_. It is clear from the figure that already near *t*_*c*_ = 0.733556, there is a clear sign of explosive growth to one giant cluster. Thus if we pick one of the nodes at random it is most likely to belong to the giant cluster and hence the corresponding microsstate is the most likely. The same is true for SR and for other values of *m* as well. Thus, it is expected that at *t* = 1 almost all the nodes will belong to one giant cluster making *μ* ≈ 1 and hence entropy at *t* = 1 should be minimally low.

**Figure 4:**
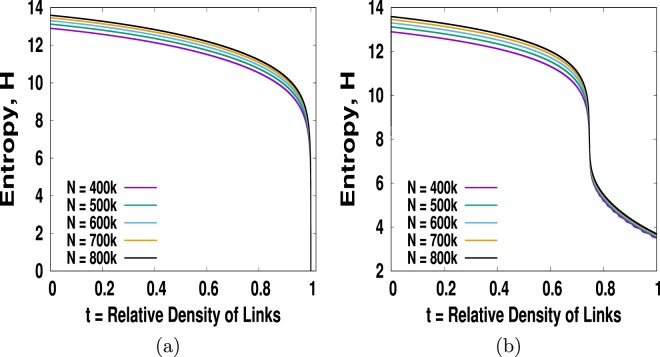
Plots entropy *H* vs *t* for *m* = 1, 3, 5 and *m* = 10. We find the critical points for *m* = 1, 3, 5 and *m* = 10 are 1, 0.7467, 0.73422 and 0.7272 respectively. The entropy plots for all *m* > 1 share the same shape except the *t*_*c*_ values decreases with increasing *m*.

Using the cluster picking probability *μ*_*i*_ in Eq. (3) we have measured entropy and shown in Fig. (4a) for *m* = 1. We see that for *m* = 1 the critical point *t*_*c*_ = 1. This is like one dimensional percolation problem where we do not have access to the other phase *t* > *t*_*c*_ since the number of links *n* for *m* = 1 cannot exceed *N* and hence *t* ≤ 1. However, as we increase the value of *m* from *m* = 1 we find that *t*_*c*_ < 1 and the *t*_*c*_ value decreases with increasing *m*. This is expected as we know that the critical point always depends on the mean connectivity which is 2 *m* for the BA network. In Fig. (4b) we show entropy versus *t* curves interpolate nicely between *t* = 0 and *t* = 1 for *m* = 3. The key features of entropy for all other *m* > 1 is the same except their *t*_*c*_ value. We all know entropy measures the degree of disorder. What is disorder in percolation anyway? Say, initially every cluster is colored with a distinct color. To do that we need *N* number different colors. The probability that a node picked at random belongs to, say green or red or any other color, is 1/*N*. Seeing a system with number of different colors as many as the number of clusters will definitely give an impression of a disordered system. Now as we keep adding links, clusters will merge to form bigger clusters. Consider that when two clusters merge the resulting cluster take the color of the larger cluster and in the case of merging two equal sized clusters the resulting cluster picks one of the two colors at random. As we continue adding links we will see a transition across *t*_*c*_ to a giant cluster of one single color that dominates the entire system. At this point, the system will look ordered in the sense that any site we pick at random is almost certain that it will belong to the giant cluster.

To get a better understanding of disorder we now focus on the frequentist version of the probabilities {*μ*_1_, *μ*_2_, ..., *μ*_*k*_}. Let us make *N* number of independent attempts to pick one node at each attempt at random with uniform probability. Say that clusters 1, 2, …, *m* are picked *n*_1_, *n*_2_, ..., *n*_*m*_ times so that ${\sum }_{i=1}^{m}\,{n}_{i}=N$. The total number of ways we could have *N* outcomes are

4 $${\rm{\Omega }}=\frac{N!}{({n}_{1})!({n}_{2})!\mathrm{....}({n}_{m})!}.$$

Taking log on either side and using *n*_*i*_ = *Nμ*_*i*_ and the Stirling’s approximation log *N*! = *N* log *N* − *N* for very large *N* we obtain

5 $$\mathrm{log}\,{\rm{\Omega }}=-N\sum _{i=1}^{m}\,{\mu }_{i}\,\mathrm{log}\,{\mu }_{i}=NH(\mu ).$$

The total entropy is therefore equal to *NH*. In the case when *n*_1_ = *n*_2_ = ... = *n*_*m*_ each cluster has the equal chance of picking means *μ*_*i*_ = 1/*N* ∀ *i* we have the most disordered state where log Ω = *N* log *N*. It means that our entropy *H* measures the average degree of ignorance or confusion associated with picking each node and answering exactly which cluster does it contain. Now as we keep adding links we will have a giant cluster of same color at *t* = 1. In this case, we are in the least confused state as we have *H*(*μ*) = 0. This is the state we regard as the most ordered phase. Thus percolation is indeed an order-disorder transition. where disorder is equivalent to degree of confusion and order is equivalent to certainty.

**Figure 5:**
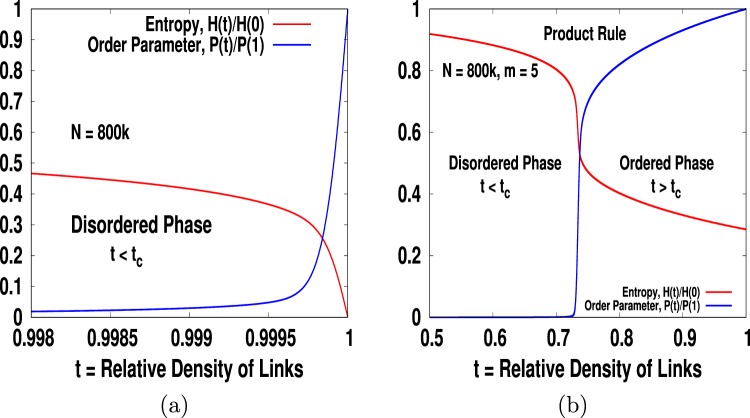
(**a**) We show plots of relative entropy *H*(*t*)/*H*(0) and relative order parameter *P*(*t*)/*P*(1) for (**a**) *m* = 1 and (**b**) *m* = 5. Both the plots are for PR only since the corresponding plots for SR have exactly the same features.

In percolation, the order parameter is defined as the relative size of the largest cluster *P* = *s*_max_/*N*. Note that at *t* ≤ *t*_*c*_ the largest cluster is miniscule in size which decreases in comparison to *N* as *N* increases and hence *P* → 0 as *N* → ∞ till *t* = *t*_*c*_. However, at above *t*_*c*_ the size of the largest cluster is proportional to the size of the system *N* itself. Thus, in the *t* > *t*_*c*_ regime *P* is non-zero and for a given value of *t* its value increases to a constant as *N* → ∞. Moreover, *P* increases sharply but without discontinuity near *t*_*c*_ and then moves slowly towards unity as *t* increases further. This is exactly how magnetization behaves with temperature during the paramagnetic to ferromagnetic phase and hence regarding *P* as the order parameter is well justified. Typically, the phase where the order parameter is always zero is regarded as the disordered phase provided entropy is significantly high there. On the other hand, the phase where entropy is minimally low but the order parameter is significantly high is called the ordered phase. We intend to present both the quantities in the same plot to see whether they compliment each other in this fashion or not. However, note that the numerical value of the maximum entropy, which is equal to log(*N*), is much higher than that of the *P*, which can at best be equal to unity. Therefore, for better comparison we plot relative entropy *H*(*t*)/*H*(0) and relative order parameter *P*(*t*)/*P*(1) in an attempt to re-scale their values so that in either cases their respective maximum values become unity. The plots of re-scaled entropy and order parameter are shown in Fig. (5a,b) for various *m* = 1 and *m* = 5 respectively for product rules only. The corresponding plots for the sum rule are not shown since they have exactly the same features except the fact that *t*_*c*_ value for *m* > 1 are different. These figures clearly show that when *P* = 0, the entropy *H* is maximally high and vice versa. It means that percolation transition, like paramagnetic to ferromagnetic transition, is accompanied by order-disorder transition.

## Specific Heat and Susceptibility

Once we know the entropy, then finding the specific heat is just a routine matter since we know its general definition

6 $$C=({\rm{control}}\,{\rm{parameter}})\times \frac{d({\rm{Entropy}})}{d({\rm{control}}\,{\rm{parameter}})}.$$

In the case of thermal phase transition, we use temperature *T* in the place of control parameter and Gibb’s or Boltzmann entropy in the place of entropy. In the case of percolation, we use *q* = (1 − *t*) as the equivalent counterpart of temperature since both entropy and order parameter behave with *q* in the similar fashion as their corresponding counterpart in the thermal phase transition. On the other hand, we use Shannon entropy as the equivalent counterpart of Gibb’s or Boltzmann entropy. The definition of specific heat for percolation therefore is

7 $$C(t)=q(t)\frac{dH}{dq(t)}.$$

**Figure 6:**
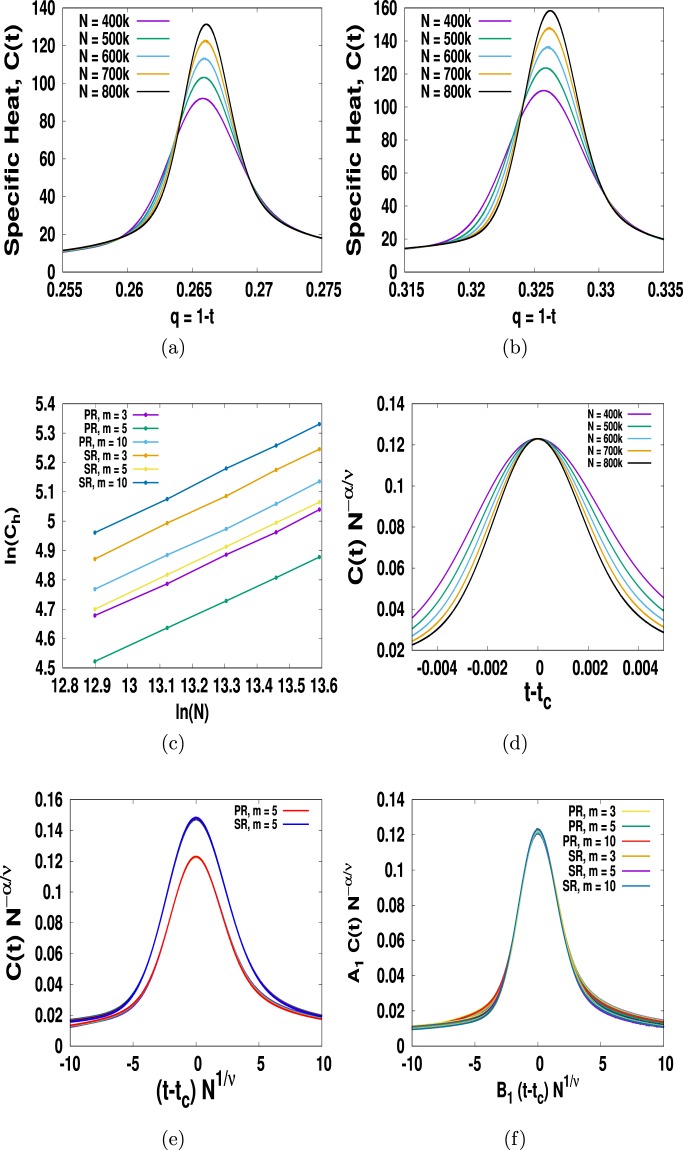
Specific heat *C* versus *t* for (**a**) PR and (**b**) SR in the BA network with *m* = 5. (**c**) The slopes of the plot log(*C*_h_) vs log(*N*), where *C*_h_ is the peak value at *t*_*c*_, for different *m* and different rules gives values of *α*/*ν*. In (**d**) we plot *CN*^−*α*/*ν*^ vs *t* − *t*_*c*_ for sum rule with *m* = 5 shows collapse of all the peaks with *α*/*ν* = 0.5119(2). We obtain the same results for SR. (**e**) We plot *CN*^−*α*/*ν*^ vs (*t* − *t*_*c*_)*N*^1/*ν*^ using 1/*ν* = 0.519577 we find that all the distinct plots in (**a**,**b**) collapse into their own scaling curve. In (**f**) we plot *A*_1_*CN*^−*α*/*ν*^ vs *B*_1_(*t* − *t*_*c*_)*N*^1/*ν*^ and find that the scaling curves for different *m* and rules collapse into a master curve for suitable metric factors *A*_1_ and *B*_1_.

Thus, differentiating *H* from first principles and multiplying the resulting value by the corresponding value of *q* = (1 − *t*), we can immediately obtain *C*(*t*). We then plot *C*(*t*) in Fig. (6a,b) as a function of *t* for PR and SR respectively and immediately see the sign of divergence at the critical point. Of course, the true divergence can only be seen in the case of the system size *N* → ∞ which is an unsurmountable limitation of the numerical simulation. One way of overcoming this is by using the finite-size scaling (FSS) hypothesis

8 $$C(t,N) \sim {N}^{\alpha /\nu }{\varphi }_{C}((t-{t}_{c}){N}^{1/\nu }),$$

where *ϕ*_*C*_(*z*) is the universal scaling function for specific heat^45^. It helps us to extrapolate the values of the critical exponents for infinite system from a set of data obtained from finite size system. One way of proving Eq. (8) is by data-collapse of distinct *C*(*t*, *N*) versus *t* for different *N* into one universal curve *ϕ*_*C*_(*z*) which is our next task.

According to Eq. (8) the peak *C*_h_ of the *C*(*t*, *N*) vs *t* curves at *t* = *t*_*c*_ increases following a power-law ${C}_{{\rm{h}}} \sim {N}^{\alpha /\nu }$. Plotting log(*C*_h_) versus log(*N*) in Fig. (6c) we find a set of parallel lines for different *m* > 1 and for different rules whose slope gives an estimate of *α*/*ν*. The same value of *α*/*ν* for all *m* regardless of product or sum rule suggest that they all share the same critical exponents. Plotting *C*(*t*, *N*)*N*^−*α*/*ν*^ versus *t*, see Fig. (6d), and finding that all the peaks collapse to one point provides a clear testament of how good is this value. We checked it for *m* = 3, 5, 10 and their product and sum rules. We find that all the peaks collapse at the same point with the same value of *α*/*ν* = 0.5119. Note that the correspondng *α*/*ν* value for EP on ER networks is 0.535. We then draw a horizontal line in the plot of *C*(*t*, *N*)*N*^−*α*/*ν*^ versus *t* and measure the distance of the intercepts of each curve from the critical point to obtain a data of *t* − *t*_*c*_ versus *N*^37,38^. Plotting this data in the log − log scale gives a straight line whose slope gives a good estimate for 1/*ν* value. Finally, we plot *CN*^−*α*/*ν*^ vs (*t* − *t*_*c*_)*N*^1/*ν*^ and after fine tuning the 1/*ν* value we obtain a perfect data-collapse with *α* = 0.98739 and 1/*ν* = 0.519577 for both the rules as shown in Fig. (6e). Furthermore we plot *A*_1_*CN*^−*α*/*ν*^ vs *B*_1_(*t* − *t*_*c*_)*N*^1/*ν*^ in Fig. (6f) with *m* = 3, 5, 10 for both the rules and find that all the scaling curves collapse into one master curve once we use appropriate metric factors *A*_1_ and *B*_1_^46^. We then use the relation $N \sim {(t-{t}_{c})}^{-\nu }$ in $C(t) \sim {N}^{\alpha /\nu }$ and find that indeed the specific heat diverges like

9 $$C(t) \sim {(t-{t}_{c})}^{-\alpha }\mathrm{.}$$

The quality of data-collapse is a clear testament of the accuracy of our *α* = 0.98739 value. Note that the *α* value of EP in BA network is slightly lower than its value in the ER network where *α* = 1.

**Figure 7:**
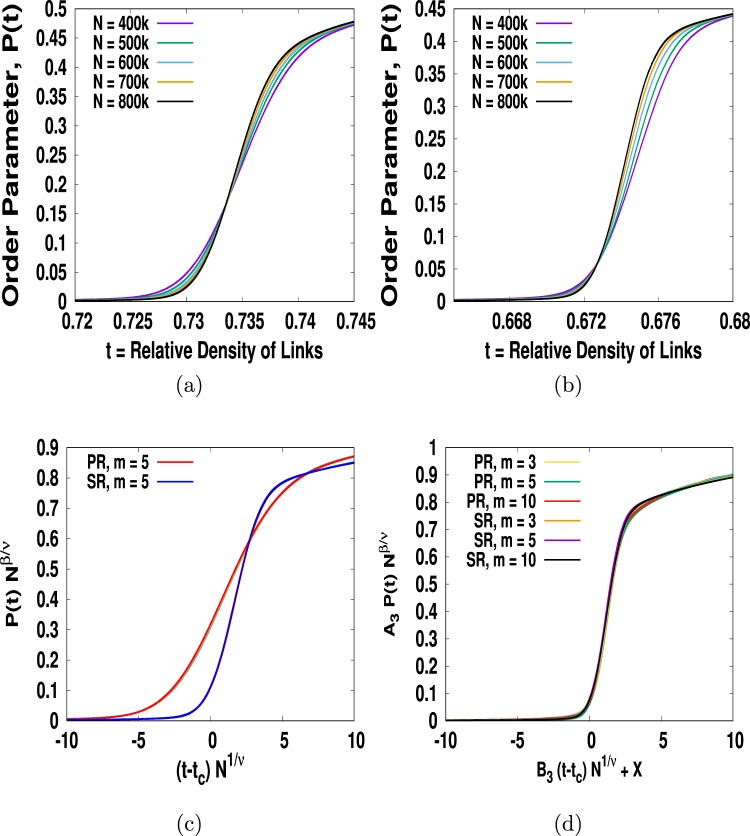
We show plots of *P* versus *t* (**a**) for both PR and (**b**) for SR. In (**c**) we plot *PN*^*β*/*ν*^ versus (*t* − *t*_*c*_)*L*^1/*ν*^ for both PR and SR. We find that all the distinct plots in (**a**,**b**) collapse into their own scaling curves sharing the same critical exponents. (**d**) Plots of *A*_2_*PN*^*β*/*ν*^ versus *B*_2_(*t* − *t*_*c*_)*L*^1/*ν*^ for PR-SR collapse into a master curve if we can choose suitable values for metric factors *A*_2_ and *B*_2_ independent of *m* > 1.

Our next goal is to find the critical exponent *β* of the order parameter. Essentially, we should find the *β* value also for infinite system for which *P* should be equal to zero over the entire regime of *t* < *t*_*c*_. This is, however, not the case as long as we work with finite size system. Plotting *P*(*t*) as a function of *t* for different system size clearly shows a sign that as *N* increases we find *P* = 0 up to an increasingly higher value of *t* as shown in Fig. (7a,b). The order parameter is said to exhibit finite-size scaling if it can be expressed as

10 $$P(t,N) \sim {N}^{-\beta /\nu }{\varphi }_{\beta }((t-{t}_{c}){N}^{1/\nu }),$$

where *ϕ*_*β*_(*x*) is the universal scaling function of *P*. Note that unlike the finite size scaling ansatz for *C*(*t*, *N*), we have a negative sign in the exponent for order parameter. It implies that if we plot *P*(*t*, *N*) vs *z* = (*t* − *t*_*c*_)*N*^1/*ν*^ then the value of *P* decreases with network size. Following the procedures in refs.^37–39^ we first find *β*/*ν* = 0.0468 and once again we obersve that its value is independent of the rules and the values of *m* except for *m* = 1. We now plot *PN*^*β*/*ν*^ vs (*t* − *t*_*c*_)*N*^1/*ν*^ with *β*/*ν* = 0.0468 and 1/*ν* = 0.5195 and find that indeed all the distinct curves of Fig. (7a,b) collapse into their respective universal curves as shown in Fig. (7c). However, even these two universal curves collapse into a master curve if we plot *A*_2_*PN*^*β*/*ν*^ vs *B*_2_(*t* − *t*_*c*_)*N*^1/*ν*^ − *X* provided we know the suitable metric factors *A*_2_, *B*_2_ and trivial shifting factor *X*. Indeed, the scaling curves for both rules and for all *m* except *m* = 1 collapse into a master curve sharing the same critical exponents as shown in Fig. (7d). It implies that their scaling functions are the same except for a multiplying metric factors *A*_2_, *B*_2_ and trivial shifting factor *X*. Substituting the relation $N \sim {(t-{t}_{c})}^{-\nu }$ in $P \sim {N}^{-\beta /\nu }$ we get

11 $$P(t) \sim {(t-{t}_{c})}^{\beta }\mathrm{.}$$

This is exactly how the order parameter behaves near critical point in the thermal CPT as well. We once again find that both PR and SR rules for all *m* > 1 share the same exponent *β* = 0.09007 which is slightly higher than that in the ER network.

Recently, we defined the susceptibility *χ*(*t*, *N*) for percolation as the ratio of the change in the order parameter Δ*P* and the magnitude of the time interval Δ*t* during which the change Δ*P* occurs. Essentially it becomes the derivative of the order parameter *P* since Δ*t* → 0 in the limit *N* → ∞ as Δ*t* = 1/*N*. Recall that susceptibility in the paramagnetic to ferromagnetic transition too is the derivative of the order parameter. Using Δ*P* = Δ*s*_max_/*N* in the definition we find

12 $$\chi (t,N)={\rm{\Delta }}{s}_{{\rm{\max }}},$$

which is essentially the jump in the largest cluster as we add link one by one. There are two distinct ways in which jump can takes place. It may happen that an already existing largest cluster ${s}_{{\rm{\max }}}^{i}$ may merge with a smaller cluster and their combined size ${s}_{{\rm{\max }}}^{f}$ will obviously be the largest cluster. Besides, it may also happen that two smaller clusters may merge such that their combined size ${s}_{{\rm{\max }}}^{f}$ may be larger than ${s}_{{\rm{\max }}}^{i}$. In either case the jump in the largest is the difference between ${s}_{{\rm{\max }}}^{f}$ and ${s}_{{\rm{\max }}}^{i}$.

**Figure 8:**
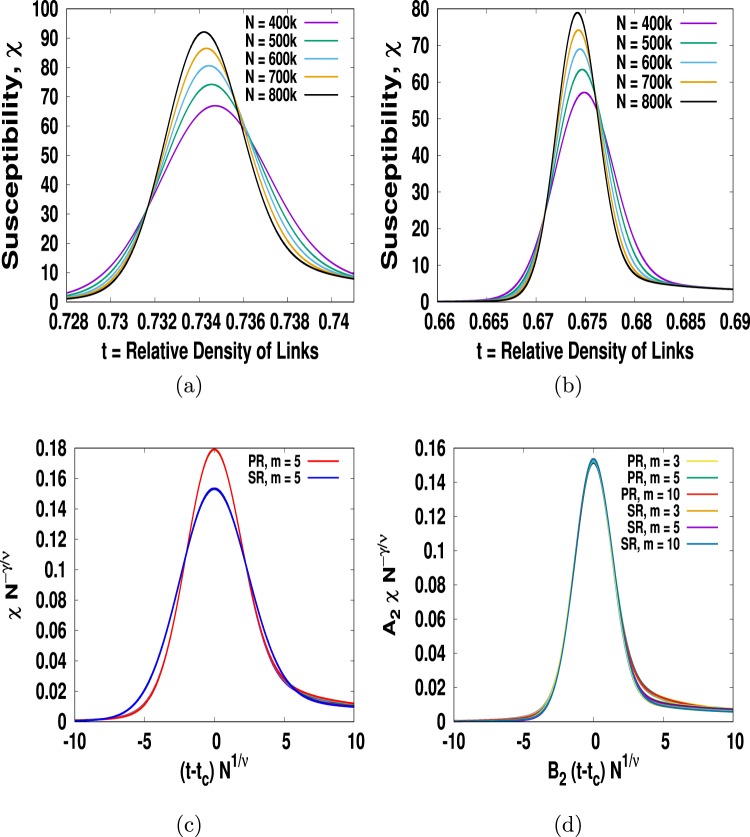
Plots of susceptibility *χ* versus *t* with *m* = 5 (**a**) for PR and (**b**) for SR. (**c**) We plot *χN*^−*γ*/*ν*^ vs (*t* − *t*_*c*_)*N*^1/*ν*^ and find distinct curves in (**a**) and (**b**) collapse into their own scaling curves. (**d**) We plot the same for PR-SR with different *m* and that distinct scaling curves collapse into a master curve if we can choose suitable metric factors *A*_3_ and *B*_3_.

Percolation theory is a statistical model and hence the numerical values of an observable from a single realization are not reproducible. We thus take the average of *χ* value over many independent realizations under the same condition and the resulting plots are shown in Fig. (8**a**) for PR with *m* = 5 as for Fig. (8**b**). This leads to assume that it too should obey the finite-size scaling form

13 $$\chi (t-{t}_{c},N) \sim {N}^{\gamma /\nu }{\varphi }_{\chi }((t-{t}_{c}){N}^{1/\nu }).$$

where *ϕ*_*χ*_(*ξ*) is known as the universal scaling function. According to Eq. (13), the susceptibility *χ*_max_ at *t* = *t*_*c*_ increases obeying a power-law ${\chi }_{h} \sim {N}^{\gamma /\nu }$ and this is true for all *m* > 1 and for both the rules. Following the same procedure as we have done for specific heat we find *γ*/*ν* = 0.4594 for both PR and for SR. We now plot *χN*^*γ*/*ν*^ vs (*t* − *t*_*c*_)*N*^1/*ν*^ in Fig. (8**c**) and find that all the distinct curves for PR and SR with *m* = 5 collapse superbly into their respective scaling curves with the same set of critical exponents *γ*/*ν* = 0.4594 and 1/*ν* = 0.5195. In Fig. (8**d**) we plot *A*_3_*χN*^*γ*/*ν*^ vs *B*_3_(*t* − *t*_*c*_)*N*^1/*ν*^ and find that all the universal scaling curves for *m* = 3, 5, 10 and for the two rules with different *m* > 1 values PR-SR belong to the same universality class regardless of the value of *m* > 1. Using now the relation $N \sim {(t-{t}_{c})}^{-\nu }$ in $\chi  \sim {N}^{\gamma /\nu }$ we find that

14 $$\chi  \sim {(t-{t}_{c})}^{-\gamma },$$

where *γ* = 0.884 for both PR and SR within the acceptable limit of error. It clearly shows that the susceptibility now diverges even without the exclusion of the largest cluster.

## Scaling and Universality

Scaling theory predicts that the various critical exponents cannot just assume values independently rather they are bound by some scaling and hyperscaling relations. One of these scaling relations is the Rushbrooke inequality where the exponents *α*, *β* and *γ* are related by

15 $$\alpha +2\beta +\gamma \ge 2.$$

Remarkably, many experiments and exactly solved models of thermal CPT suggest that Eq. (15) holds more as equality than as inequality which is also supported by static Widom scaling^47^. Substituting our values from the Table 1 in Eq. (15) we find *α* + 2*β* + *γ* = 2.05 which clearly suggests that the Rushbrooke relation holds exactly as an inequality and approximately as an equality. Earlier we found the same results for EP on ER network and RP on square and weighted planar stochastic lattice (WPSL)^32,33,38^. These studies clearly suggest the robustness of the Rushbrooke relation in percolation inependent of whether explosive or random percolation is done on networks or on lattice.

**Table 1:** The critical exponents and Rushbrooke inequality for explosive percolation in BA and ER networks.

Skeleton	*ν*	*α*	*β*	*γ*	*α* + 2*β* + *γ*
BA	1.946	0.987	0.090	0.884	2.051
ER	1.869	1.00	0.084	0.893	2.061

The classification of second order phase transition under different physical conditions into universality classes is yet another interesting proposition. It has been put forth by Kadanoff in 1970 who suggested that two or more systems sharing the same values of critical exponents and scaling functions are said to belong to the same universality class. It is well-known that the critical exponents of RP on spatially embedded lattice are universal in the sense that their values depend only on the dimension of the lattice and independent of whether it is site or bond type percolation. However, there exists just one exception as we have recently found namely that RP on WPSL does not belong to the universality class of two dimensional lattices^32,33,37^. The only way WPSL is different from all the known two dimensional lattices is that its coordination number distribution follows a power-law. Now the question is: What happens when the skeleton is infinite dimensional network? It is already known that RP on ER and on the Bethe lattice belongs to the same universality class owing to the fact that both of them are infinite dimensional skeletons^48^. Radicchi and Castellano on the other hand reported that the site-bond universality is violated even in the case of RP on network^49^. Earlier Cohen *et al*. showed that RP on scale-free network, albeit infinite dimensional, does not belong to the universality class of RP on ER and Bethe lattice^34^. Thus the scale-free nature of the skeletons are different from the rest as far as random percolation is concerned.

Explosive percolation, on the other hand, has been proposed only in 2009 and we witnessed a furry of activities for at least up to the first three years in order to resolve the issue of whether EP describes continuous or discontinuous phase transition. There has hardly been any attempt since then to classify EP into universality classes. Only recently, we have shown that explosive percolation of PR and SR type in the ER network belongs to the same universality class. Earlier two groups, Bastas *et al*. and Yong *et al*., reported that site and bond type explosive percolation on the same lattice do not belong to the same universality class^29,50^. The present work is about EP on scale-free networks and we once again find that PR and SR belong to the same universality class but it is different from the PR-SR universality class of EP on ER. It is, however, noteworthy to mention that the corresponding exponents are close but they can in no way be equal. Finding the PR-SR universality in two systems as different as the BA and ER networks suggests that PR-SR universality is quite robust. It appears that the absence of usual site-bond universality in RP is being replaced by PR-SR universality in EP^38^.

## Conclusions

We have used a class of BA networks where each node is born with *m* links and shown that its properties are significantly different for different *m* values albeit the exponent of the degree distribution remains the same. We have used such networks as skeleton to study explosive percolation for both product and sum rule of the Achlioptas process. Our first goal was to see the role of *m* and also to see the role of the product and sum rule in fixing the critical points and the critical exponents. Our first observation is that the critical point *t*_*c*_ decreases systematically with increasing *m* starting at *t*_*c*_ = 1 for *m* = 1. We have then shown that the critical exponents remain the same for all *m* > 1 independent of product and sum rules. It implies that EP on the BA network belongs to one grand universality class. Interestingly, the PR-SR universality class of EP on the BA networks is different from that of EP on ER network although the difference in the values of their critical exponents in some cases is quite slim. It suggests that PR-SR universality in explosive percolation is quite robust and it appears as if they are the equivalent counterpart of the much known bond-site universality of RP on spatially embedded lattices.

Our second goal was to understand what order and disorder means in percolation and find out if percolation in general and explosive percolation in particular is an order-disorder transition. To this end, we have found that the behaviour of entropy and order parameter is reminiscent of what we see in the paramagnetic to ferromagnetic transition. It suggests that explosive percolation is indeed an order-disorder transition. Another potentially interesting finding is that the critical exponents *α*, *β*, *γ* obey the Rushbrooke inequality. Earlier, we have shown that it also holds for EP on ER networks. These results suggest that no matter how the values of the critical exponents change they still always obey the Rusbrooke inequality but always close to equality. So far, we have studied EP on scale-free and random networks. It would be interesting to see if the small-world nature of the network topology has any effect in determining the universality class and in obeying the Rushbrooke inequality. In our future endeavour, we intend to study EP in scale-free networks via mediation-driven attachment process since this has a wide range of exponent 2 < *λ* ≤ 3^36^. It would be interesting to see how much impact the exponent of the degree distribution has in fixing the critical exponents *vis-a-vis* the universality class.
